# Effects of Nigella sativa on VCAM-1 and ICAM-1: A systematic review of preclinical and clinical studies

**DOI:** 10.34172/jcvtr.025.33343

**Published:** 2025-03-18

**Authors:** Zeinab Faghfoori, Zeinab Javadivala, Aida Malek Mahdavi

**Affiliations:** ^1^Food Safety Research Center (salt), Semnan University of Medical Sciences, Semnan, Iran; ^2^Department of Nutrition, School of Nutrition and Food Sciences, Semnan University of Medical Sciences, Semnan, Iran; ^3^Department of Health Education & Promotion, Faculty of Health, Tabriz University of Medical Sciences, Tabriz, Iran; ^4^Connective Tissue Diseases Research Center, Tabriz University of Medical Sciences, Tabriz, Iran

**Keywords:** *Nigella sativa*, Vascular cell adhesion molecule-1, Intercellular adhesion molecule-1

## Abstract

The objective of present review was to assess all studies about effect of *Nigella sativa* (*N. sativa*) on vascular cell and intercellular adhesion molecules-1 (VCAM-1 and ICAM-1) under different situations. Search was performed until May 2024 using Scopus, PubMed, Web of Science, and Google Scholar databases without any restriction and alert services were utilized following the primary search. The references cited in related papers were also evaluated. Nineteen studies including human (n=4), animal (n=11), and *in vitro* (n=4) were eligible. All *in vitro* and majority of animal researches were indicative of the favorable effects of *N. sativa* and thymoquinone in attenuating VCAM-1 and ICAM-1 levels; however, three animal studies did not show any significant effect. Results of clinical trials were conflicting. In two clinical trials, supplementation with *N. sativa* oil and *N. sativa* powder led to significant reduction in VCAM-1 levels in coronary artery disease (CAD) and Hashimoto’s thyroiditis patients, whereas no significant change occurred according to the other clinical trial involving subjects with the risk factor for cardiovascular disease (CVD). Furthermore, significant reduction in ICAM-1 levels occurred after *N. sativa* oil consumption in two clinical trials involving type 2 diabetic and CAD patients, whilst no significant change was noticed in subjects with the risk factor for CVD and Hashimoto’s thyroiditis patients. *N. sativa* seems beneficial in attenuating VCAM-1 and ICAM-1 levels under different situations; however, additional long-term controlled clinical trials are needed for making concise conclusions about the effect of *N. sativa* on endothelial dysfunction related biomarkers.

## Introduction

 Endothelial injury has a main function in expansion of vascular events including atherosclerosis, and is characterized by low-grade inflammation that initiates upregulation of cell adhesion molecules.^1^ Upregulation of various adhesion molecules induces inflammatory pathways and results in a chronic inflammatory situation, if not being managed appropriately.^2^ Therefore, dysregulation of adhesion molecules can cause different inflammatory and immune-related diseases.^2^ Adhesion molecules are glycoproteins on cellular surfaces responsible for the connection between cells or between the extracellular matrix and cells.^3^ During the preliminary phases of atherosclerosis, these molecules enable monocytes to stick to the endothelial cells and migrate under endothelium after attaching to the injured endothelial cells.^4^ Vascular cell adhesion molecule-1 (VCAM-1) and intercellular adhesion molecule-1 (ICAM-1) are amongst the basic contributing adhesion molecules in atherosclerosis.^5^ VCAM-1 and ICAM-1 are generated on stimulated endothelial cells and atherosclerosis-prone locations leading to the accumulation of inflammatory monocytes in the endothelium.^3^ Therefore, inhibiting VCAM-1 and ICAM-1 can delay atherosclerosis development and has a significant role in the prevention of atherosclerosis.

 Recently, herbal compounds with potential antioxidant and anti-inflammatory activities have been reported to beneficially affect vascular endothelium and endothelial function.^6^ Thus, considering plants in treating conditions associated with endothelial injury and dysfunction has become an area of interest.^7^ One such medicinal herb is *Nigella sativa* (*N. sativa*).

*N. sativa*, usually recognized as black cumin or black seed, is a rich source of antioxidants and bioactive agents including polyphenols, flavonoids, saponins, alkaloids, proteins, fatty acids, vitamins, and minerals.^8^ Thymoquinone (TQ) is the most active component found plentifully in *N. sativa*, together with its derivatives like thymol and thymohydroquinone.^8^ *N. sativa* is a traditional herb, which is a member of the Ranunculaceae family and is frequently consumed in the Middle East, Western Asia, North Africa, and Eastern Europe.^8^ Antioxidant, anti-inflammatory, hypotensive, hypoglycemic, and hypolipidemic activities have been related to* N. sativa*.^9^ In addition, *N. sativa* and its constituents have shown beneficial effects against diabetes mellitus, hypertension, obesity, dyslipidemia, and metabolic syndrome^9^, which are linked with vascular dysfunction. Therefore, amelioration in any of these diseases can also improve vascular action.

 The effects of *N. sativa* on adhesion molecules specifically VCAM-1 and ICAM-1 have been studied in recent years; however, some discrepancies exist among the results. *In vitro* studies^10-13^ as well as some animal^14-20,21^ and clinical^25-27^ studies indicated the beneficial effects of *N. sativa* in reducing VCAM-1 and ICAM-1, whilst in other animal^22-24^ and clinical^26,28^ researches, no effect and/or increased concentrations of these adhesion molecules were reported. Given the inconsistent findings of previous related studies and lack of any comprehensive systematic review encompassing clinical and preclinical studies altogether in this field, present systematic review was conducted to evaluate the effects of *N. sativa* on VCAM-1 and ICAM-1 under different situations considering data from clinical, animal, and *in vitro* models.

## Methods

###  Study protocol and search strategy

 This study agrees with the Preferred Reporting Items for Systematic Reviews and Meta-Analyses (PRISMA) guidelines.^29^ The protocol of study was registered with the International Prospective Register of Systematic Reviews (PROSPERO) under the registration number CRD42024556099.

 A literature search was conducted utilizing electronic databases, encompassing Web of Science, Scopus, PubMed, and Google Scholar, up to May 2024. We also utilized search alerts to notice relevant articles after the primary search. The terms utilized to search within titles, abstracts, and keywords were: “Nigella sativa”, “Nigella sativas”, “sativa, Nigella”, “Cumin, Black”, “Black Cumin”, “Black Cumins”, “Cumins, Black”, Kalonji, Kalonjus, “thymoquinone”, dihydrothymoquinone, “2-isopropyl-5-methylbenzoquinone”, “2-methyl-5-isopropyl-p-benzoquinone”, “Vascular Cell Adhesion Molecule-1”, “Vascular Cell Adhesion Molecule 1”, “Inducible Cell Adhesion Molecule 110”, INCAM-110, “Vascular Cell Adhesion Molecule”, VCAM-1, “CD106 Antigen”, “Antigen, CD106”, “Antigens, CD106”, “CD106 Antigens”, “Intercellular Adhesion Molecule-1”, “Intercellular Adhesion Molecule 1”, ICAM-1, “CD54 Antigen”, “Antigen, CD54”, “Antigens, CD54”, “CD54 Antigens”. We utilized both MeSH terms and text words without inflicting any restrictions on language or publication date. Two researchers separately performed the search and screening activities. Duplicates were recognized and eliminated. The references of relevant papers were assessed to uncover related studies. A consensus existed between the two authors on article selection, and any potential discrepancies were addressed and resolved by the third researcher.

###  Inclusion and exclusion criteria

 This scientific review adhered to strict criteria to choose relevant studies on the effects of *N. sativa* on VCAM-1 and ICAM-1. Studies were included if they assessed the effects of *N. sativa* compared with a control arm and if the full text of the research was accessible. Conversely, studies excluded from the review were those that did not directly assess the impact of *N. sativa* on VCAM-1 and ICAM-1, such as review articles, book chapters, or studies on the combined impact of *N. sativa* with other compounds. Additionally, any research not published in a peer-reviewed journal or with limited access to the full text was excluded.

###  Data extraction

**Figure 1:**
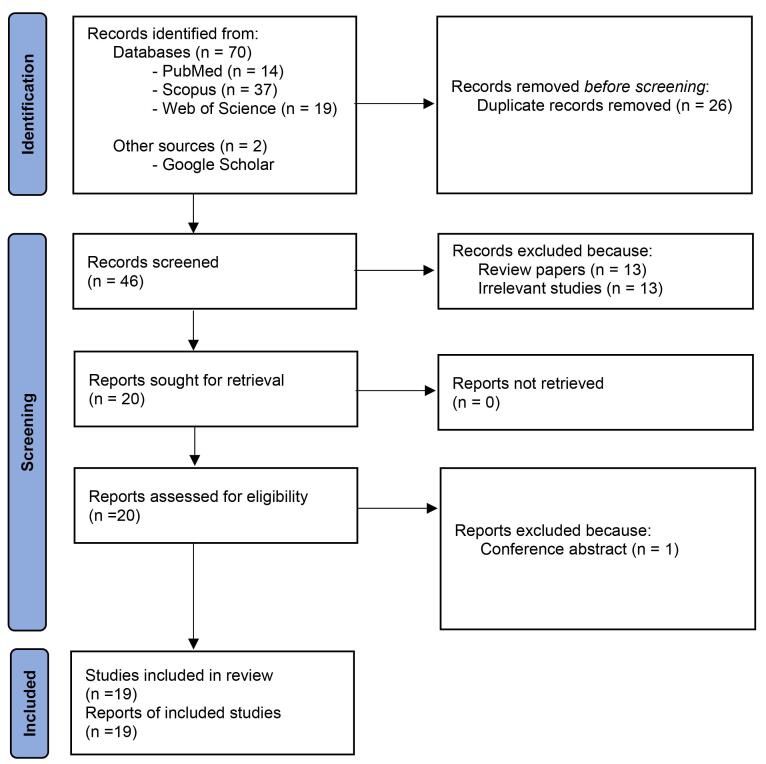
The flowchart of screening and selecting articles

 After using inclusion and exclusion criteria, 19 studies were selected for further analysis (Figure 1). Data pertaining to the first author’s last name, issue year, sample characteristics, type and dose of *N. sativa* administered, period of intervention, and reported outcomes were gathered from the selected studies. A comprehensive overview of these studies is presented in Tables 1 to 3.

**Table 1:** Characteristics of included *in vitro* studies

**Author **	**Population**	**Intervention**	**Dose**	**Duration**	**Findings **
Khan et al^10^	Human coronary artery endothelial cells	*Nigella sativa* oil and Thymoquinone	55, 110, 220, and 440 µg/ml 4.5, 9.0, 18.0, and 36.0 µm	24 hours	Significant dose-dependent reduction in VCAM-1 and ICAM-1 gene and protein expressions
Huwait et al ^11^	Human THP-1 macrophages	Thymoquinone	2.5, 5, 7.5, and 10 µM	24 hours	Significant decrease in mRNA expression of ICAM-1
Umar et al^12^	RA synovial fibroblasts	Thymoquinone	1–5 μM	2 hours	Significant dose-dependent reduction in ICAM-1 and VCAM-1 expression
Xuan et al ^13^	Mouse dendritic cells	Thymoquinone	1, 5, 10, and 20 μM	24 hours	Significant inhibition of ICAM-1 expression

VCAM-1, vascular cell adhesion molecule-1; ICAM-1, intracellular adhesion molecule-1; RA, rheumatoid arthritis.

**Table 2:** Characteristics of included animal studies

**Author**	**Population**	**Intervention**	**Dose**	**Route**	**Duration**	**Findings**
Triastuti et al ^14^	Rats with sub-chronical cigarette smoke exposure (n = 50)	Ethanolic extract of *Nigella sativa*	0.3, 0.6, and 1.2 g/kg/day	Gastric tube	4 weeks	Significant dose-dependent reduction in VCAM-1 expression compared to the exposed control group
Ashour et al^15^	Rats with renal ischemia-reperfusion injury (n = 48)	Thymoquinone	10 mg/kg/day	Intravenous	10 days	Significant reduction in renal VCAM-1 level compared to the renal ischemia-reperfusion injury control group
Ashour et al^16^	Rats with remote hepatic injury post-renal reperfusion (n = 30)	Thymoquinone	10 mg/kg/day	Intravenous	10 days	Significant reduction in liver VCAM-1 level compared to the reperfusion injury control group
Alzohairy et al ^17^	Rats with *Benzo(a)pyrene*-induced lung injury (n = 32)	Thymoquinone	50 mg/kg/day	Oral	8 weeks	Significant reduction in ICAM-1 level compared to the lung injury control group
Abbasnezhad et al ^18^	Streptozotocin-induced diabetic rats (n = 70)	Hydroalcoholic extract of* Nigella sativa *seed	100, 200, and 400 mg/kg/day	Gavage	6 weeks	Significant reduction in VCAM-1 mRNA expression compared to the diabetic control group
Alhusaini et al^19^	Rats with sodium fluoride-induced acute renal injury (n = 50)	Thymoquinone	10 mg/kg/day	Oral	4 weeks	Significant reduction in protein expression of VCAM compared to the acute renal injury control group
Elmorsy et al ^20^	Experimentally-induced atherosclerosis rabbits (n = 30)	*Nigella sativa *powder	150 mg/kg/day	Gavage	20 weeks & 8 weeks	Significant reduction in serum VCAM-1 and ICAM-1 levels compared to the atherosclerosis control group
Saleh and El-Abhar^21^	Shistosoma mansoni infected mice (50-55)	Thymoquinone	5 and 10 mg/kg/day	Oral	2 weeks	Significant reduction in ICAM-1 expression in Kupffer and inflammatory cells compared with the infected control group
Al-Asoom et al^22^	Rats with physiological cardiac hypertrophy (n = 45)	*Nigella sativa *suspension	800 mg/kg/day	Oral	8 weeks	No significant change in serum ICAM-1 level compared with the control group
Al Wafai et al^23^	Streptozotocin-induced diabetic rats (n = 150)	*Nigella sativa *aqueous extractand *Nigella sativa* oil and Thymoquinone	2 mL/kg/day 0.2 mL/kg/day 5 mg/kg/day	Intra-peritoneal injection	6 days/week	No significant change in ICAM-1 mRNA expression in pancreatic tissue compared with the diabetic control group
Finlay et al^24^	Wild-type (WT), Neu4 KO (Neu4 knockout), and Neu1-CathA KD (Neu1 deficient and cathepsin A deficient) mice (n = 15)	Thymoquinone	2.5 mg	Intra-peritoneal injection	-	Significant increase in serum ICAM-1 compared with the control group

VCAM-1, vascular cell adhesion molecule-1; ICAM-1, intracellular adhesion molecule-1.

**Table 3:** Characteristics of included human studies

**Author**	**Population**	**Intervention**	**Dose**	**Route**	**Duration**	**Findings**
Tavakoli-Rouzbehani et al ^25^	Coronary artery disease patients (n = 60)	*Nigella sativa *oil	2 g/day	Oral	8 weeks	Significant decrease in serum VCAM-1 and ICAM-1 levels compared with the coronary artery disease control group
Abbasalizad Farhangi & Tajmiri^26^	Hashimoto's thyroiditis patients (n = 40)	*Nigella sativa *powder	2 g/day	Oral	8 weeks	(1) Significant decrease in serum VCAM-1 levels compared with the control group (2) No significant change in serum ICAM-1 levels compared with the Hashimoto's thyroiditis control group
Elgarf et al^27^	Type 2 diabetic patients (n = 56)	*Nigella sativa *oil	1.8 g/day	Oral	12 weeks	Significant decrease in serum ICAM-1 levels compared with the diabetic control group
Emamat et al^28^	Subjects with at least one risk factor for cardiovascular disease (n = 50)	*Nigella sativa *oil	1 g/day	Oral	8 weeks	No significant change in plasma VCAM-1 and ICAM-1 levels compared with the control group

VCAM-1, vascular cell adhesion molecule-1; ICAM-1, intracellular adhesion molecule-1.

###  Evaluation of bias risk 

 The risk of bias (RoB) in the included clinical, animal, and *in vitro* researches was assessed using the Cochrane Collaborationʼs tool, the Systematic Review Centre for Laboratory Animal Experimentation (SYRCLE’s) RoB tool, and the Checklist for Reporting *In vitro* Studies (CRIS) instruction, respectively. The SYRCLEʼs RoB tool relies on the Cochrane Rob tool and both of the tools have six domains, and every domain was judged as possessing a low, unclear, or high risk.^8^

## Results

###  Study Selection

 Overall 72 papers were detected primarily (Figure 1). After removing duplicates, 46 papers were screened by their titles and abstracts. Finally, out of 20 potentially related papers, one paper was deleted due to abstract in conference. Finally, 19 papers including human (n = 4), animal (n = 11), and *in vitro* (n = 4) researches were kept. Tables 1 to 3 show details of the researches.

###  Characteristics of the included studies

 The primary characteristics of the included studies are outlined in Tables 1 to 3. Studies were conducted in the following countries: Malaysia,^10^ Saudi Arabia,^11,16,17,19,22^ USA,^12^ Germany,^13^ Indonesia,^14^ Egypt,^15,20,21,27^ Iran,^18,25,26,28^ Lebanon,^23^ Canada.^24^ The included *in vitro* studies used TQ with different doses, ranging from 1 to 36 μM. In one *in vitro* study,^10^ *N. sativa* oil with doses of 55, 110, 220, and 440 µg/ml was also used. The duration of treatment was 24 hours in three *in vitro* studies^10,11,13^ and 2 hours in one *in vitro* study.^12^ A variety of *in vitro* models were used including human coronary artery endothelial cells,^10^ human THP-1 macrophages,^11^ RA synovial fibroblasts,^12^ and mouse dendritic cells.^13^ Moreover, the included animal studies varied in length, ranging from 6 days/week to 20 weeks and route of administration including oral, gastric tube, gavage, intravenous, and intra-peritoneal injection. Ethanolic extract of *N. sativa *with doses of 0.3, 0.6, and 1.2 g/kg/day and hydro-alcoholic extract of *N. sativa* with doses of 100, 200, and 400 mg/kg/day were used in two animal studies,^14,18^ respectively. In the other animal studies, *N. sativa* powder with a dose of 150 mg/kg/day,^20^ TQ with doses of 5 to 50 mg/kg/day^15-17,19,21,23^ and 2.5 mg,^24^ *N. sativa* suspension with a dose of 800 mg/kg/day,^22^ *N. sativa* aqueous extract with a dose of 2 mL/kg/day as well as *N. sativa* oil with a dose of 0.2 mL/kg/day^23^ were used. A variety of animal models were studied in these animal investigations including rats with sub-chronical cigarette smoke exposure,^14^ rats with renal ischemia-reperfusion injury,^15^ rats with remote hepatic injury post-renal reperfusion,^16^ rats with *Benzo(a)pyrene*-induced lung injury,^17^ streptozotocin-induced diabetic rats,^18,23^ rats with sodium fluoride-induced acute renal injury,^19^ experimentally-induced atherosclerosis rabbits,^20^ Shistosoma mansoni-infected mice,^21^ rats with physiological cardiac hypertrophy^22^ as well as Wild-type, Neu4 knockout, and Neu1 deficient and cathepsin A deficient mice.^24^ Furthermore, the included clinical trials varied in length, ranging from 8 to 12 weeks. *N. sativa* oil^25,27,28^ were used in three studies with doses from 1 to 2 g/day. In the other clinical trial,^26^ *N. sativa* powder was used with dose of 2 g/day. A variety of patient populations were studied in the included clinical trials. This includes subjects with coronary artery disease,^25^ Hashimoto’s thyroiditis,^26^ type 2 diabetes,^27^ and subjects with at least one cardiovascular disease (CVD) risk factor^28^.

###  In vitro investigations 

 Four *in vitro* studies were eligible (Table 1). Khan et al^10^ indicated that 55, 110, 220, and 440 µg/ml *N. sativa* oil and 4.5, 9.0, 18.0, and 36.0 µm TQ for 24 hours led to remarkable dose-dependent decrease in VCAM-1 and ICAM-1 gene and protein expressions in human coronary artery endothelial cells. In another study, Huwait et al^11^ stated that 2.5, 5, 7.5, and 10 µM TQ for 24 hours significantly decreased mRNA expression of ICAM-1 in human THP-1 macrophages. Furthermore, Umar et al^12^ showed that 1–5 μM TQ for 2 hours caused significant dose-dependent reduction in expression of ICAM-1 and VCAM-1 in RA synovial fibroblasts. Xuan et al^13^ also demonstrated that 1, 5, 10, and 20 μM TQ for 24 hours significantly inhibited ICAM-1 expression in mouse dendritic cells.

###  Animal investigations 

 Eleven animal studies were eligible (Table 2).Triastuti et al^14^ indicated that 0.3, 0.6, and 1.2 g/kg/day ethanolic extract of *N. sativa* for 4 weeks dose-dependently reduced VCAM-1 expression in rats with sub-chronical cigarette smoke exposure compared to the cigarette smoke-exposed control rats. Ashour et al^15,16^ showed that 10 mg/kg/day TQ for 10 days considerably reduced renal and liver VCAM-1 level in rats with renal ischemia-reperfusion injury and in rats with remote hepatic injury post-renal reperfusion, respectively compared with the reperfusion injury controls. Moreover, Alzohairy et al^17^ reported that oral consumption of 50 mg/kg/day TQ for 8 weeks significantly alleviated ICAM-1 level in rats with *Benzo(a)pyrene*-induced lung injury compared to the lung injury control ones. Furthermore, Abbasnezhad et al^18^ reported that 100, 200, and 400 mg/kg/day hydro-alcoholic extract of* N. sativa* seed for 6 weeks considerably reduced VCAM-1 mRNA expression in streptozotocin-induced diabetic rats compared with the diabetic control animals. Alhusaini et al^19^ demonstrated that 10 mg/kg/day TQ for 4 weeks significantly reduced protein expression of VCAM in rats with sodium fluoride-induced acute renal injury compared to the acute renal injury control group. Elmorsy et al^20^ found that 150 mg/kg/day *N. sativa* powder led to remarkable reduction in serum VCAM-1 and ICAM-1 levels in experimentally-induced atherosclerosis rabbits in comparison to the atherosclerosis control arm. In addition, Saleh and El-Abhar^21^ suggested that 5 and 10 mg/kg/day TQ for 2 weeks significantly reduced ICAM-1 expression in Kupffer and inflammatory cells in Shistosoma mansoni-infected mice compared with the infected controls. Another research by Al-Asoom et al^22^ showed that 800 mg/kg/day *N. sativa* suspension for 8 weeks did not cause significant change in serum ICAM-1 level in rats with physiological cardiac hypertrophy compared with the control arm. In another study, Al Wafai et al^23^ indicated that 2 mL/kg/day* N. sativa* aqueous extract, 0.2 mL/kg/day *N. sativa* oil, and 5 mg/kg/day TQ for 6 days/week did not significantly change in ICAM-1 mRNA expression in pancreatic tissue in streptozotocin-induced diabetic rats in comparison with the diabetic controls. Finlay et al^24^ suggested that 2.5 mg TQ significantly increased serum ICAM-1 in Wild-type, Neu4 knockout, and Neu1 deficient and cathepsin A deficient mice compared to the control mice.

###  Clinical investigations 

 Four clinical studies were eligible (Table 3).According to Tavakoli-Rouzbehani et al^25^ reported that 2 g/day *N. sativa* oil for 8 weeks considerably decreased serum VCAM-1 and ICAM-1 in coronary artery disease patients compared with the control patients. Also, Abbasalizad Farhangi and Tajmiri^26^ reported that consuming 2 g/day *N. sativa* powder for 8 weeks caused significant decrease in serum VCAM-1 levels, whereas did not significantly alter serum ICAM-1 levels in Hashimoto’s thyroiditis patients compared with the controls. Furthermore, Elgarf et al^27^ indicated that oral consumption of 1.8 g/day *N. sativa* oil for 12 weeks remarkably decreased serum ICAM-1 levels in patients with type 2 diabetes compared with the diabetic control subjects. In another study, Emamat et al,^28^ consuming 1 g/day *N. sativa* oil for 8 weeks did not remarkably change plasma VCAM-1 and ICAM-1 levels in subjects with at least one CVD risk factor compared to the control subjects.

###  Methodological quality 

**Figure 2:**
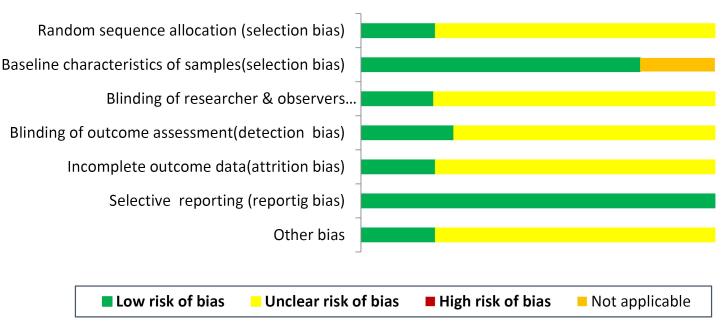
The risk of bias assessment across the studies (n = 19)

 There was an unclear risk for selection bias (absence of data about the randomization procedure: n = 15); detection bias (masking of outcome evaluation: n = 14); performance bias (masking of the researcher regarding intervention: n = 15) and attrition bias (n = 15). Reporting bias: n = 19 and baseline details of animal and *in vitro* samples: n = 15 was low. A report for risk of bias was noted in Figure 2.

## Discussion

 To the authors’ knowledge, this systematic review is the first assessing the existing literature regarding the effect of *N. sativa* on VCAM-1 and ICAM-1 under different situations considering data from clinical, animal, and *in vitro* researches. All* in vitro*^10-13^ and almost all animal^14-20,21^ investigations demonstrated the favorable effects of *N. sativa* and TQ in attenuating VCAM-1 and ICAM-1 levels; however, three animal studies^22-24^ did not demonstrate any significant effect of *N. sativa* on VCAM-1 and ICAM-1 levels. Results of clinical trials regarding the effect of *N. sativa* on VCAM-1 and ICAM-1 were also conflicting.^25-28^ Some clinical trials^25,26^ showed that *N. sativa* oil and *N. sativa* powder supplementation led to significant reduction in VCAM-1 levels, whereas no significant change was observed in VCAM-1 levels according to Emamat et al^28^ research. Furthermore, significant decrease in ICAM-1 was observed after *N. sativa* oil consumption in studies by Tavakoli-Rouzbehani et al^25^ and Elgarf et al,^27^ while no significant alteration was noticed in ICAM-1 according to some other clinical trials.^26,28^ Variations in study designs, characteristics of study samples, baseline concentrations of VCAM-1 and ICAM-1, preparation and administration methods, dosage, duration, and bioavailability rate of *N. sativa* and/or its components seem to be responsible for discrepancies among researches. The* N. sativa* or its active constituents were directly utilized in animal models, whereas in clinical trials, *N. sativa* powder and oil were prescribed in the form of capsule. Furthermore, level of TQ, the principle compound of* N. sativa*, varies according to the preparation procedure and storage condition of products of* N. sativa*, which can lead to meaningful variety in bioactive compounds among studies. Altogether, this review indicated that adhesion molecules such as VCAM-1 and ICAM-1 had a meaningfully descending orientation after using *N. sativa *and/or TQ. This study was consistent with Mohebbatia and Abbasnezhad^30^ study, which concluded that *N. sativa* and TQ had a protective impact on endothelial dysfunction initiated by diabetes. However, a newly published meta-analysis of controlled trials concluded that supplementation with* N. sativa* did not have a meaningful impact on the endothelial function responses including ICAM-1 and VCAM-1 in subjects with CVD or the risks of CVD, highlighting a disagreement that emphasizes the intricacy of *N. sativa *effects on endothelial function and the need for further research.^31^ These authors also noted that the pooled data were severely heterogeneous, which affected their results.^31^

 As mentioned earlier, upregulation of cellular adhesion molecules like VCAM-1 and ICAM-1 has a close relationship with endothelial injury and dysfunction, which is the primitive phase of atherosclerosis. Therefore, VCAM-1 and ICAM-1 can be considered as predictors of CVD events and closely related to the atherosclerosis development.^32^ *N. sativa* is a well-tolerated and safe herb and most of its helpful medicinal properties are due to volatile oil, of that TQ is a main component.^33^ Different mechanisms are indicated for potential ameliorative effect of* N. sativa* or its bioactive agent TQ on adhesion molecules. *N. sativa* has a lipid-lowering property, which is due to the suppression of *de novo* cholesterol synthesis or induction of bile acid excretion. Therefore, *N. sativa* through modulating lipid profile may inhibit VCAM-1 gene expression, reduce atherosclerotic plaque generation, and promote endothelial integrity.^34-36^ Moreover, *N. sativa* may attenuate VCAM-1 and ICAM-1 concentrations via decreasing the expression of oxidized low-density lipoprotein receptor-1 (LOX-1), which is the principle receptor in endothelial cells for oxidized low-density lipoprotein (LDL).^18^ Oxidized LDL uptake by LOX-1 reduces endothelial nitric oxide synthase (eNOS) expression and nitric oxide production and further induces adhesion molecule expression, thereby leading to endothelial dysfunction.^37,38^ Moreover, oxidized LDL binding to LOX-1 contributes to oxidative stress and superoxide generation and nuclear factor-kappa B (NF-κB) induction.^39^ The other mechanism is that *N. sativa* may decrease adhesion molecules’ expression via reducing reactive oxygen species (ROS) generation and endothelial cell injury by its direct antioxidant feature.^40^ A part of the antioxidant activity of *N. sativa *is attributed to its TQ content. This compound increases the bioavailability of nitric oxide through stimulating the activity of the enzyme eNOS, which is important for vasodilation and vascular health.^41,42^ The antioxidant function of *N. sativa *has also been demonstrated via increasing the antioxidant enzymes.^22^ The impaired antioxidant system can increase ROS and pro-inflammatory mediators leading to elevated adhesion molecule expression and atherosclerosis development.^43^ Additionally, TQ in *N. sativa* can lower adhesion molecules’ expression via suppressing pro-inflammatory cytokines or enzymes such as monocyte chemoattractant protein-1, tumor necrosis factor-α, interleukin (IL)-1β, IL-8, IL-6, nuclear factor-kappa B, and cyclooxygenase-2, which lead to anti-inflammatory effect in the body.^44^

 A limitation of the current review was the limited number of clinical studies, whereas the number of preclinical investigations was acceptable. The strength of current review was that all preclinical and clinical investigations were gathered in a systematic manner without any restriction regarding language and/or publication date.

## Conclusion

 In conclusion, *N. sativa* seems beneficial in attenuating VCAM-1 and ICAM-1 levels under different situations. This systematic review was just a description of accessible literature about the influence of *N. sativa* on VCAM-1 and ICAM-1 together with potential mechanisms and indicated the need for additional long-term controlled clinical trials to make concise conclusions about the effect of *N. sativa* on endothelial dysfunction related biomarkers.

## Competing Interests

 The authors declare no conflict of interest.

## Ethical Approval

 This study was approved by the Ethics Committees of Tabriz University of Medical Sciences, Tabriz, Iran.
